# Nummular headache: a case report of remission following ketogenic diet and botulinum toxin type A injections

**DOI:** 10.3389/fneur.2023.1200907

**Published:** 2023-05-19

**Authors:** Yan Tereshko, Simone Dal Bello, Christian Lettieri, Enrico Belgrado, Giovanni Merlino, Gian Luigi Gigli, Mariarosaria Valente

**Affiliations:** ^1^Clinical Neurology Unit, Department of Neuroscience, Udine University Hospital, Udine, Italy; ^2^Neurology Unit, Department of Neuroscience, Udine University Hospital, Udine, Italy; ^3^Department of Medicine (DAME), University of Udine, Udine, Italy

**Keywords:** nummular headache, ketogenic diet, botulinum toxin, pain, rare disorder

## Abstract

Nummular headache is an unusual facial pain disorder with no evidence-based therapy recommendations. The ketogenic diet is an alternative therapy that demonstrated to be effective in migraineurs, but it was never used in the setting of nummular headache. We describe a 58-years old female patient with nummular headache successfully treated with a 6-months ketogenic diet and botulinum toxin type A injections. Ketogenic diet could be an effective alternative/complementary therapy in nummular headache patients although more studies are needed to confirm our results.

## Introduction

Nummular headache is a rare primary headache, first described in 2002 (1), characterized by focal pain localized on the surface of the head in a small coin-shaped or elliptical area; this painful area has typically well-defined borders and no underlying lesions (2, 3). This disorder is mostly focal, and any region of the head could be affected although it's slightly more common in the *tuber parietale* of the parietal scalp and on the right side and tends to affect extra-trigeminal territories (4, 5). Multifocal, synchronous or asynchronous, painful areas have been described and, in some cases, the painful area extends across the midline (6). The pain, mild to moderate in intensity, is often described as stabbing or oppressive and, less commonly, throbbing, sharp, or even burning (7). Exacerbations can be triggered by mechanical stimulation or can be spontaneous (2). Patients often lament allodynia, paresthesia, and/or hypoesthesia restricted to the symptomatic area and autonomic signs are typically absent although trophic changes are described (1, 8). This disorder is more common in women, with spontaneous onset in the mid-forties (4). Most cases are chronic, and the pain could be persistent, intermittent, or fluctuating. Cases of spontaneous remission, or pseudo-remissions after treatment (when the pain is replaced by discomfort), are described in the literature (7). Since nummular headache is a rare disorder with only case series and no large studies in literature, there are no evidence-based recommendations regarding the therapy (9); moreover, the ketogenic diet was never used in this setting. Here we describe a case of nummular headache successfully treated consecutively with Ketogenic Diet and Botulinum Toxin type A injections.

## Case presentation

On January 2020, a 58-years old female patient came to our headache outpatient clinic complaining of moderate (mean NRS 6/10) persistent pain in the vertex and nearby right parietal scalp. The onset of the headache, which was spontaneous, was on the 12^th^ of November 2019. The background pain was described as oppressive, with fixed and defined borders, ellipsoidal shape, and a 5–6 cm diameter. The patient denied nausea, vomiting, phonophobia, photophobia, or osmophobia. Physical activity did not influence the symptoms while stressful events worsened or exacerbated the pain; she reported daily exacerbations (NRS 9/10) with throbbing quality. This disturbance was partially responsive to paracetamol. She denied previous head trauma or infections. Her medical history comprehended asthma, hypothyroidism, arterial hypertension, and obesity; she had familiarity for migraine on her mother's side. She had also multiple allergies (acetylsalicylic acid, ibuprofen, mites, kiwi, strawberries, and aspartame). The neurological examination was unremarkable apart from allodynia in the site of the nummular headache. Head CT scan, brain MRI, and blood work-up were normal. Initially, she was diagnosed as New Daily Persistent Headache; the diagnosis was later changed to nummular headache based on the clinical features of her headache based on the ICHD-3 criteria (10). She started preventive therapy with amitriptyline 20 mg with no benefit. 3 months after the initial evaluation, she started venlafaxine 150 mg/day with slight benefit on intensity (NRS 9/10), but with no effect on the frequency of exacerbations of the headache or on the background pain, which remained persistent. Due to the efficacy of the dietary approach with the ketogenic diet on migraine (11), we decided to apply this dietary regimen to our case, although there are no cases in the literature that support its use on nummular headache. Since our patient had a BMI of 34.2, we opted for the Very-Low-Calorie Ketogenic Diet (VLCKD) that she started in November 2021 and concluded in May 2022. Her 730 Kcal diet consisted of a daily intake of 85 grams of proteins, 30 g of fats, and 30 g of carbohydrates. Her exacerbations and background pain improved both at 3-months and 6-months evaluations after diet's initiation (Table 1). Her BMI improved from 34.2 Kg/m^2^ to 26.7 Kg/m^2^ and her Fat Mass reduced from 34.8 Kg to 18.2 Kg. Ketonemia or urinary ketone levels were not monitored during the diet. The dietary intervention was well-tolerated by the patient, with no adverse effects.

**Table 1:** The trend in pain during the two treatments.

	**Exacerbation pain NRS**	**Exacerbations frequency days/month**	**Background pain NRS**
**VLCKD therapy**			
Baseline	9	31	6
3-month	2	6	2
6-month	4	2	2
**BoNT/A therapy**			
Baseline (July 2022)	7	4	5
1° BoNT/A session	0	0	2
2° BoNT/A session	0	0	2
3° BoNT/A session	0	0	0

**Figure 1:**
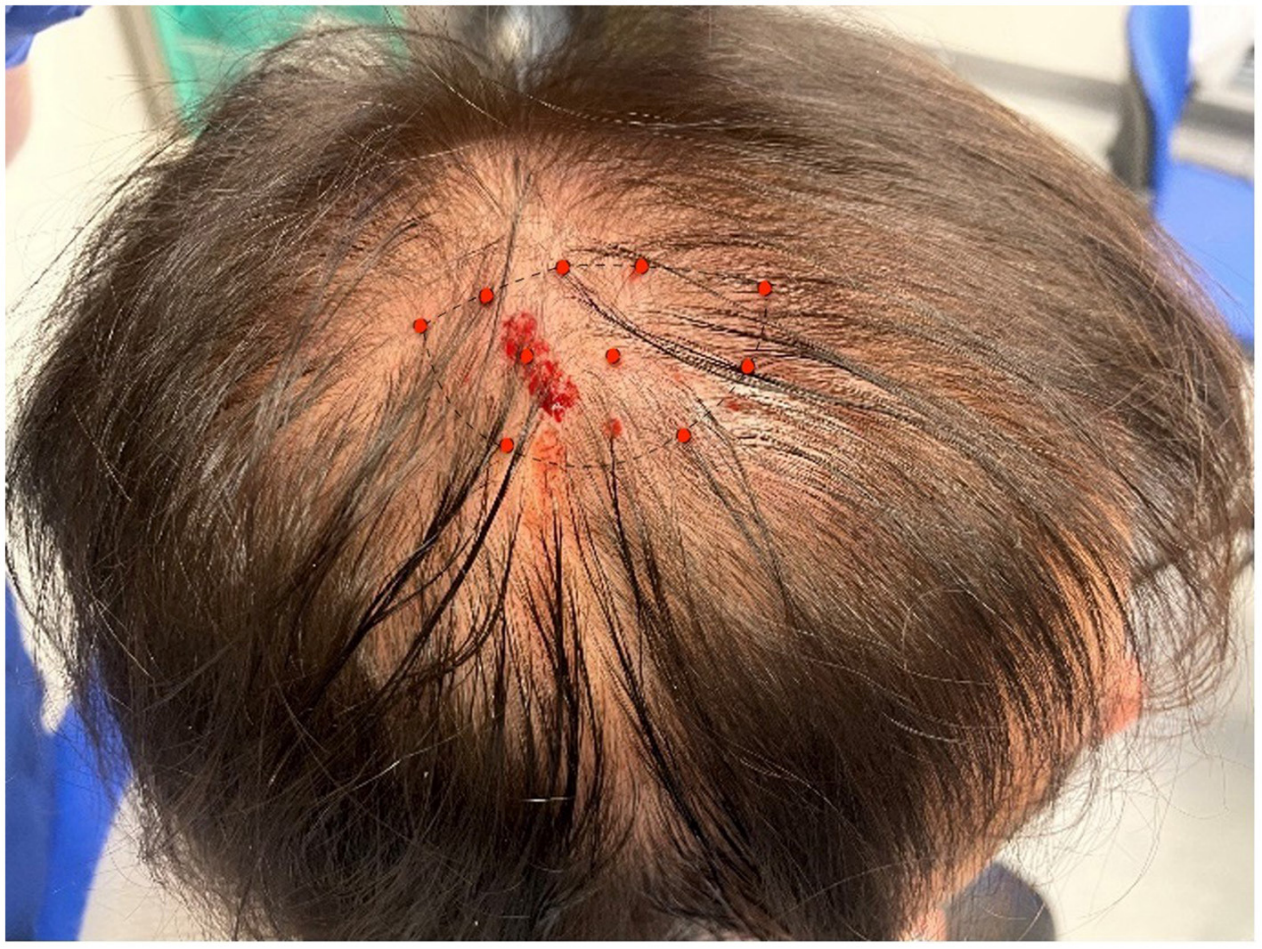
The location of the nummular headache of our patients. The borders are represented by the dashed line; the red dots indicate the sites of subcutaneous BoNT/A injections (5 U per site).

In June 2022, the VLCKD was discontinued, as planned, and the patient started a normocaloric hypoglucidic diet (1,400 Kcal/day). Although no rebound weight gain was observed, the nummular headache gradually became more severe. In July 2022, we decided to treat the patient with BoNT/A (Botulinum Toxin type A) injections in the nummular headache location at a total dose of 50 U. In each treatment, a vial of BoNT/A was diluted with 1 ml of 0.9% sodium chloride (100 U/ml dilution). The treatment was performed with subcutaneous injections in the ellipsoidal-shaped area of pain, with 5 U per site and a total of 10 points (Figure 1). The injections were performed using a 30-gauge 0.3 mm × 8 mm needle. Treatment was performed three times with an interval time between one round and another of 3–4 months, since the duration of the effect was the same; the mean onset of the BoNT/A effect was 10 days after the injections. Her exacerbation pain was abolished, and background pain and allodynia gradually disappeared; the patient reported only slight discomfort located in the treated area. No adverse effects were reported. We evaluated the frequency and intensity of the exacerbations as well as the intensity of the background pain (Table 1).

## Discussion

Nummular headache is a rare disorder, often underdiagnosed due to its rarity and the diversity of its clinical presentation. The incidence of this disorder is 6.4/100.000 per year (7) and the etiology is still unknown, but some authors suggest that it might be due to sensory dysfunction of a terminal branch of a pericranial nerve, configuring an unusual form of neuralgia; other authors hypothesize that nummular headache is a localized form of complex regional pain syndrome or epicrania. Other authors suggested a central mechanism because anesthetic nerve blockade was ineffective and the pain area could cross the midline (4). Young age, female sex, and early diagnosis are favorable predictors of response to therapy (9). Due to the rarity of this disorder, with only single case reports or limited case series, there are no evidence-based recommendations regarding the therapeutic approach. Nummular headache responds to NSAIDs and indomethacin (12) although some case series reported no clear improvement (13, 14). Gabapentin, pregabalin, carbamazepine, oxcarbazepine, topiramate, amitriptyline, metoprolol, valproate, lamotrigine, flunarizine were used as a preventive therapy with conflicting results (9, 12–14). In our patient, the nummular headache did not improve with amitriptyline while venlafaxine was slightly effective only on headache intensity, still not setting up a form of headache refractory to oral treatments given the failure of only two lines of therapy (15). Transcutaneous Electrical Nerve Stimulation (TENS), anti-CGRP monoclonal antibodies, and subcutaneous peripheral nerve field stimulation (3, 9, 12, 16–20) have also been used with some positive results. Lidocaine was reported to be ineffective (1, 13). Botulinum toxin type A has been used in this setting with encouraging, although partial, results: in 2008 Mathew et al. (21) described four patients with nummular headache treated with significant improvement with 25 U of BoNT/A in the affected area. Dusitanond et al. (22) treated 5 patients with a mean dose of 16 U reporting improvement in only three, while Ruscheweyh et al. (23) reported only one that responded to BoNT/A. However, the details were not reported in both studies. In 2018, Martins et al. reported 8 cases of nummular headache with excellent response while, in 2019, García-Azorín et al., treated 53 patients with BoNT/A in an open-label, non-randomized, prospective study and reported a significant reduction in both headache frequency and intensity. There are different approaches of botulinum toxin administration for nummular headache, those who prefer to inject the toxin in 4–6 sites, distributed in form of a cross (5, 24), and those who, as in our case adopt a circumferential approach, with injections sites around the perimeter of the painful area. The latter approach is aimed to targeting epicranial nerve endings (21), whose dysfunction is considered a possible pathogenic event, able to triggering nummular headache (23). Further studies should be conducted in order to test this hypothesis. In our case report, the patient was able to achieve remission with BoNT/A subcutaneous injections at a dose of 50 U, thus positively testing a higher dose than reported so far in the literature. Moreover, before BoNT/A injections, our patient underwent VLCKD treatment with significant improvement in nummular headache background pain, pain exacerbations and in frequency. KD is an effective therapeutic option in migraine (11) however, there are no studies regarding its effect on nummular headache. The analgesic mechanisms underlying the functioning of the ketogenic diet are not known at present, although it may be determined by its ability to reduce neuronal hyperexcitability, typical of both chronic pain and epilepsy, conditions in which such a diet is applied, acting through multiple mechanisms (25) with a probably key role related to the activation of adenosine receptors (26). Moreover, weight loss seems to be correlated with the reduction of migraine frequency and intensity (27). Due to its long-lasting effect (7 months) and the worsening of the condition after the scheduled interruption of KD, a placebo effect of the diet is unlikely, although impossible to rule out. We suppose that the improvement of nummular headache with VLCKD was due to its anti-inflammatory action and its ability to reduce neuronal hyperexcitability. Weight loss may have been also beneficial, but headache severity turned to increase with the end of VLCKD, although no rebound weight gain was observed.

## Conclusion

There are no evidence-based data regarding the therapy for nummular headache. In our case report, KD therapy significantly improved background pain, exacerbation pain, and frequency. BoNT/A injections were able to achieve remission in our patient. If confirmed by further observations, KD could become a complementary approach and a possible alternative to the injections of onabotulinum toxin A, although the last one remains a more effective and long-term compatible option. In conclusion, the ketogenic diet could be part of the therapeutic armamentarium for patients with nummular headache, including pharmacological, dietary, and topic therapies such as botulinum toxin, to be considered especially in drug-resistant forms.

## Data availability statement

The original contributions presented in the study are included in the article/supplementary material, further inquiries can be directed to the corresponding author.

## Ethics statement

Ethical review and approval was not required for the study on human participants in accordance with the local legislation and institutional requirements. The patients/participants provided their written informed consent to participate in this study. Written informed consent was obtained from the participant/patient(s) for the publication of this case report.

## Author contributions

Writing—original draft preparation: YT and SD. Writing—review and editing: MV and GG. Supervision: CL, GM, and EB. All authors have read and agreed to the published version of the manuscript.

## Clinical implications

New therapeutic perspectives for the resolution of a disorder that, although rare, turns out to be particularly disabling.
